# Comparative SEM/EDS analysis of gunshot residue from non-toxic and traditional ammunitions employed by Dubai police

**DOI:** 10.3389/fchem.2025.1670901

**Published:** 2025-12-02

**Authors:** Asma M. Askar, Roudha A. Alblooshi, Hamda A. Alobeidli, Rashed H. Alremeithi, Iltaf Shah

**Affiliations:** 1 General Department of Forensic Science and Criminology, Dubai Police GHQ, Dubai, United Arab Emirates; 2 Department of Chemistry, College of Sciences, United Arabs Emirates University, Al Ain, United Arab Emirates

**Keywords:** forensic science, gunshot residue (GSR), inorganic gunshot residue (IGSR), non-toxic ammunition (NTA), heavy metal free (HMF) ammunition, SEM/EDS

## Abstract

Analysis and identification of gunshot residue (GSR) are considered critical forensic evidence in shooting incident investigations. This study comparatively analyzed gunshot residue from Fiocchi non-toxic ammunition (NTA), ADCOM, and NATO ammunitions, all commonly utilized by Dubai Police, using Scanning Electron Microscopy coupled with Energy Dispersive X-ray Spectroscopy (SEM/EDS) following the American Society for Testing and Materials (ASTM) E1588-20 standards. The elemental compositions of ammunition components, including cartridge cases, bullets, gunpowder, and primers, were thoroughly characterized. Primer residue revealed significant elemental differences, with Fiocchi NTA ammunition unexpectedly containing detectable lead particles, although at lower levels compared to ADCOM and NATO ammunition. Across all ammunition types, GSR particles predominantly measured below 3 μm, effectively differentiating them from common environmental contaminants. GSR particle deposition was consistently higher on shooters’ dominant right hands due to firearm mechanics and hand dominance. Particle counts generally decreased over time post-discharge but were influenced significantly by shooter activities rather than elapsed time alone. Notably, limitations within the ASTM E1588-20 classification scheme resulted in no identifiable Heavy-Metal-Free (HMF) GSR particles for Fiocchi NTA, emphasizing the need for updated and expanded classification criteria. Future research is recommended to enhance forensic methods and classification frameworks to accommodate evolving ammunition formulations.

## Introduction

Gunshot residue (GSR) are the unburnt and partially burnt gaseous particles released upon the discharge of a firearm. They comprise a complex mixture of organic and inorganic particles that originate from the primer, gunpowder, cartridge case, lubricants used and any metals from the firearm (Gorey et al., 2024; Karahacane et al., 2019).

When the trigger is pulled, the firearm’s hammer strikes the primer located at the back of the cartridge case, igniting it. The resulting combustion generates intense heat and pressure, vaporizing primer chemicals to form inorganic gunshot residue (IGSR), which mixes with the gases from the propellant gunpowder to form organic gunshot residue (OGSR). IGSR are composed mainly of heavy metals such as lead (Pb), barium (Ba), and antimony (Sb), while OGSR mainly includes volatiles and semi-volatile organic compounds such as diphenylamine and nitroglycerin (Romanò et al., 2020; Wenceslau et al., 2025).

As the bullet exits from the cartridge, it carries with it these organic and inorganic residue, along with other materials from the bullet, cartridge case, and the firearm itself. Afterwards, these residues adhere primarily to the shooter’s clothes and hands, being near to the firearm. Additionally, as the air currents disperse these particles, they settle on nearby surfaces and individuals. The spread of these residues is extensive as they remain airborne for some time before fully settling. Contact with GSR-contaminated clothes or skin can facilitate transfer to secondary objects. The quality and quantity of gunshot residue are influenced by factors including the type of firearm and bullet, environmental conditions, and the chemical burn rate (Brożek-Mucha, 2017; Rosengarten et al., 2021; Shaw, 2019).

These GSR can provide high-value information in forensic investigations since they are scattered in the place and time of a gunshot scene. Thus, GSR can be collectable evidence to link between an individual with the shooting incident. In addition to that, they may determine the firing distance, ammunition used, distinguish between entrance and exit wound and may differentiate between a potential homicide, accidental shooting and suicide among the rest (Brożek-Mucha, 2017; Doña-Fernández et al., 2018).

Historically, forensic detection of IGSR has focused on characteristic spherical particles composed of Pb, Ba, and Sb, typically emitted from traditional, toxic ammunition primers (Doña-Fernández et al., 2018). However, due to health concerns from lead exposure and environmental contamination, newer lead-free primers have emerged (Costa et al., 2016; El Khoury Moussa et al., 2024). These alternative primers often contain silicon (Si), aluminum (Al), zinc (Zn), sulfur (S), titanium (Ti), potassium (K), and sometimes copper (Cu), resulting in GSR particles with distinct elemental profiles and morphologies (El Khoury Moussa et al., 2024; Maitre et al., 2017; Romanò et al., 2020). Such particles, typically less dense, non-spherical, and consisting of low atomic-number elements, pose detection challenges under traditional forensic standard (Maia et al., 2022). Consequently, distinguishing between toxic and non-toxic GSR requires careful attention to elemental profiles and particle morphology, as well as adjustments to detection thresholds and interpretive criteria. In forensic contexts, misidentification or failure to detect non-toxic GSR may lead to false exclusions or underreporting of firearm discharge, especially when relying solely on Pb–Ba–Sb criteria. Recent studies, such as those by Romanò et al., emphasize the need to recognize unique markers of non-toxic residue—such as Al–K–Si or Ti–Zn–S signatures—to ensure accurate forensic conclusions (Romanò et al., 2020).

Forensic analysis of GSR employs a suite of complementary techniques to detect and characterize both inorganic and organic residue. Scanning Electron Microscopy with Energy-Dispersive X-ray Spectroscopy (SEM-EDS) remains the forensic main standard for IGSR—offering non-destructive, high-resolution imaging and elemental analysis to identify characteristic Pb–Ba–Sb particles according to ASTM E1588 (Charles and Jonckheere, 2022; Nunziata, 2019; Seyfang et al., 2019). However, other analytical techniques have been explored for specific forensic applications. María López-López et al. demonstrated the capability of Laser-Induced Breakdown Spectroscopy (LIBS) for elemental analysis of gunshot residue (GSR), showing its effectiveness in visualizing GSR distribution patterns for estimating shooting distances and providing compositional information. However, this methodology was found insufficient for single-particle identification compared to SEM-EDS (López-López et al., 2017). Xiang Li et al. successfully applied Inductively Coupled Plasma Mass Spectrometry (ICP-MS) for analyzing GSR particles contaminated by blood stains, highlighting ICP-MS’s applicability in complex forensic scenarios (Li et al., 2024). Courtney Vander Pyl et al. investigated Gas Chromatography–Mass Spectrometry (GC-MS) for detailed elemental quantification of OGSR and concluded that GC-MS alone is insufficient and should be combined with complementary analytical methods such as Liquid Chromatography–Mass Spectrometry (LC-MS/MS) (Vander Pyl et al., 2023). Recent reviews have emphasized the importance of integrated multi-technique approaches, including SEM-EDS, LIBS, MS-based methods, and electrochemical analyses, to ensure comprehensive and reliable detection of GSR (Gorey et al., 2024; Jain and Yadav, 2021).

Based on the standard protocol proposed by the ASTM E1588-20, the criteria for identifying a particle as ‘‘characteristic’’ with SEM/EDS is according to the elemental composition (Pb–Ba–Sb) and morphology (spheroidal shape). The other particles can only be classified as ‘‘consistent with GSR’’ or “commonly associated with GSR” as it shows in Table 1. Due to emerging non-toxic ammunition (NTA), new classes of particles have appeared, with unique elemental compositions now recognized as characteristic or consistent under revised forensic standards (ASTM E1588-20, 2020; Charles et al., 2022; Nunziata, 2019; Seyfang et al., 2019).

**TABLE 1:** Modern classification of GSR particles composition detected with a SEM/EDS as described in ASTM E1588–20.

Characteristic of GSR	Consistent with GSR	Commonly associated with GSR	Lead-free characteristic of GSR	Lead-free consistent with GSR
Lead–barium–antimony (Pb–Ba–Sb)	Lead, barium, calcium, silicon (Pb-Ba-Ca-Si) Barium, calcium, silicon. (Ba-Ca-Si) Antimony, barium (Sb-Ba) Lead, antimony (Pb-Sb) Barium, aluminum (Ba-Al) Lead, barium (Pb-Ba)	Lead (Pb) Antimony (Sb) Barium (Ba)	Gadolinium–Titanium–Zinc (Gd–Ti–Zn) Gallium–Copper–Tin (Ga–Cu–Sn)	Titanium–Zinc (Ti–Zn) Strontium (Sr)

It has also been reported that variability between primer lots, including within the same manufacturer, and firearm “memory effects” may introduce compositional differences in recovered residues (Burnett and Nunziata, 2023). Furthermore, recent studies have highlighted the limitations of SEM/EDS, where instrument parameters such as pixel size and resolution strongly influence the probability of detecting characteristic IGSR particles (Onetto et al., 2022). These factors emphasize the need for cautious interpretation of GSR results and refinement of current analytical frameworks.

The primary aim of this study was the comprehensive characterization and analysis of gunshot residue originating from both toxic and emerging non-toxic ammunition types currently utilized by Dubai Police. Analyses were conducted using SEM-EDS following ASTM E1588-20 standard, examining variables such as sample collection from the shooter’s right and left hands, elapsed intervals after discharge (0, 1, 2, and 4 h), and the number of shots fired (1, 3, and 5).

## Materials and methods

### Shooting experiments

**FIGURE 1:**
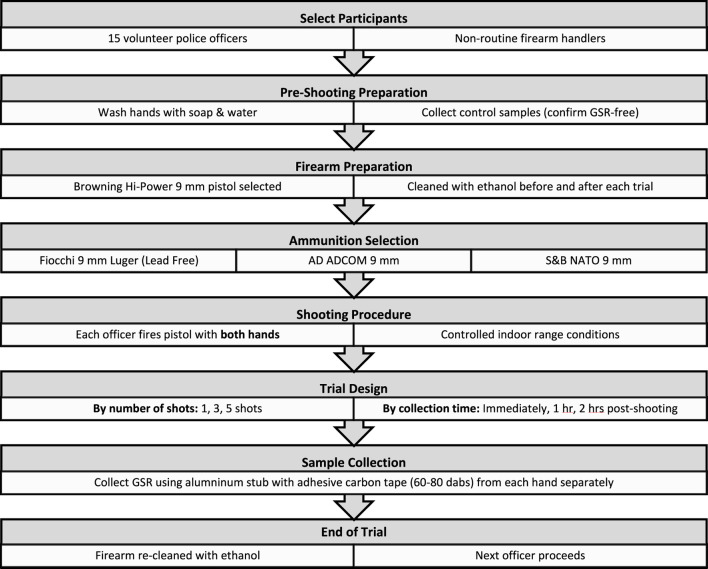
Experimental design and workflow for the shooting trials.

Fifteen volunteer police officers participated in the shooting trials conducted at the Dubai Police Forensic Laboratories’ indoor range. All participants were non-routine firearm handlers. Strict anti-contamination controls were applied: prior to each session, volunteers washed their hands with soap and water, and control samples were collected to confirm the absence of gunshot residue (GSR). A Browning Hi-Power 9 mm semi-automatic pistol was used throughout the study, and three types of ammunition were tested (9 mm Luger Fiocchi, 9 mm AD ADCOM, and 9 mm S&B NATO). To prevent cross-contamination, the pistol was thoroughly cleaned with ethanol before and after each trial. Each officer fired the pistol with both hands under consistent indoor conditions. Samples were collected across different firing sequences (1, 3, and 5 shots) and post-shooting intervals (immediately, 1 h, and 2 h). Two separate hand samples (right and left) were collected for each experimental trial, yielding a total of 54 samples across 27 trials. The overall experimental workflow is illustrated in Figure 1. A detailed experimental matrix summarizing volunteers, ammunition types, number of shots, time points, and per-hand collections is provided in Appendix Table A1.

### Sample collection and preparation

**FIGURE 2:**
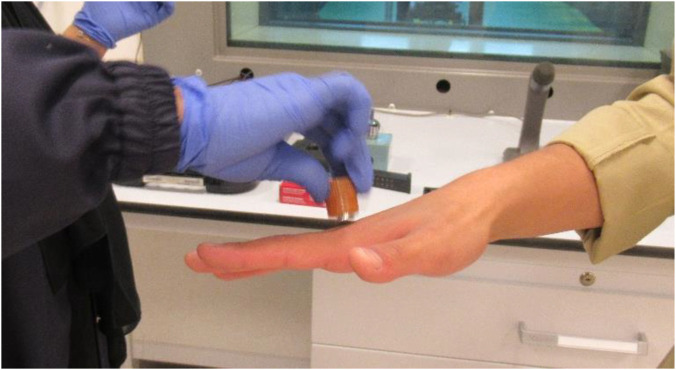
Sample collection procedure: GSR particles being collected from a volunteer’s hand using an aluminum stub coated with carbon tape adhesive.

Gunshot residue (GSR) sampling was performed using double-faced adhesive carbon tape (Micro to Nano, Product no.: 15-000412) affixed to aluminum SEM stub (TED PELLA, Product no.: 16111). The adhesive surface was systematically dabbed 60–80 times across the volunteers’ hands (Figure 2), ensuring thorough coverage of both dorsal and palmar regions, with particular emphasis on the thumb and index finger areas, commonly associated with firearm handling. Separate stubs were designated for the left and right hands to maintain sampling integrity, in line with previously validated protocols (Charles et al., 2022; Goudsmits et al., 2019; Reis et al., 2003).

### Scanning Electron Microscopy with Energy Dispersive X-ray spectroscopy (SEM/EDS)

A COXEM CX-200 Plus scanning electron microscope equipped with an Oxford EDS instrument was utilized for analyzing GSR samples. Ammunition parts were characterized using standard EDS elemental analysis, while GSR samples required analysis using specialized AZTEC GSR software (Version 5.0 SP1).

### Ammunition parts analysis using EDS

Elemental analysis was conducted on all components of the ammunition, including the casing, bullet, gunpowder, and primer, for each selected ammunition type (Fiocchi NTA, ADCOM, and NATO). All ammunition types were obtained from a single manufacturing lot, and lot/serial numbers were recorded. Analysis parameters included a working distance of 16 mm, a spot size of 12 nm, and an acceleration voltage of 15 keV, performed under secondary electron (SE) image mode.

### GSR analysis using AZTEC GSR software

GSR samples on stubs were directly transferred into the SEM chamber using forceps, which were cleaned and confirmed to be free of contamination, without further preparation. Samples were analyzed under backscattered electron (BSE) image mode with a working distance of 16 mm, spot size of 12 nm, and acceleration voltage of 20 keV.

The AZTEC GSR software performed automated searches for all particle combinations required by ASTM E1588-20. The stub area was segmented into rectangular fields for analysis, with the number and size of fields determined by the applied magnification. An image magnification of ×500 was used, resulting in approximately 1,100–1,300 fields, a BSE image scan size of 1,024 pixels, and a feature detection size of 2 pixels (0.62 µm). Calibration was conducted using internal standards (copper and rhodium) to establish the BSE signal range for bright particle detection. EDS was subsequently performed to determine elemental compositions, which were verified manually against the characteristic particles as defined by ASTM E1588-20, (2020). A GSR standard was used as positive control, and GSR analysis was performed automatically by the AZTEC GSR software.

## Results and discussions

### Ammunitions characterization

Each ammunition type and its components (cartridge case, bullet, gunpowder, and primer residue) were systematically analyzed by SEM/EDS to compare their elemental composition.

**FIGURE 3:**
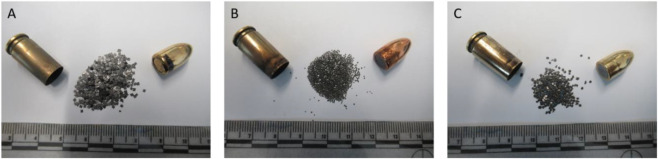
Cartridge case, bullet, gun powder and primer residue on the cartridge case of **(A)** Fiocchi NTA **(B)** ADCOM ammunition **(C)** NATO ammunition.

**FIGURE 4:**
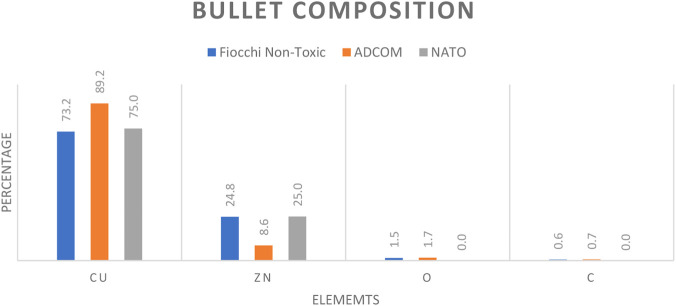
Bullet composition of Fiocchi NTA, ADCOM and NATO ammunitions.

**FIGURE 5:**
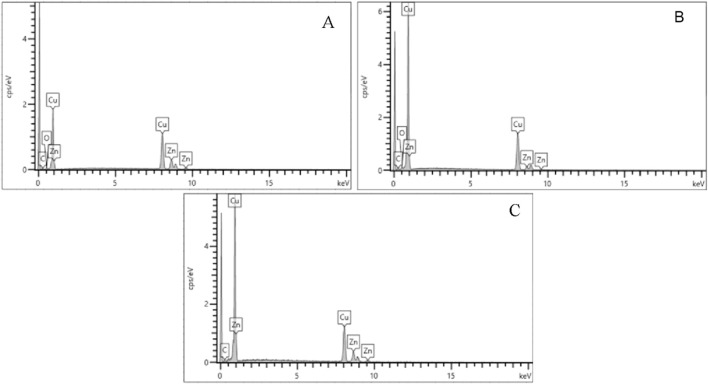
Energy-dispersive X-ray (EDS) spectra of the elemental composition of bullets from **(A)** Fiocchi NTA **(B)** ADCOM ammunition **(C)** NATO ammunition.

The ADCOM bullet exhibited the highest copper content, reaching ∼90% Cu, which correlates with its distinctive bronze coloration (Figures 3B, 4). In contrast, bullets from Fiocchi NTA and NATO contained lower copper levels, averaging ∼75% Cu, consistent with their relatively lighter, yellowish metallic appearance (Figures 3A,C). This ∼15% difference is forensically significant since bullet jacket composition influences both residual formation and ballistic characteristics. The accompanying EDS spectra (Figure 5) confirms that copper is the dominant element across all bullets, while small alloying differences in Zn may serve as discriminating features when comparing ammunition types.

**FIGURE 6:**
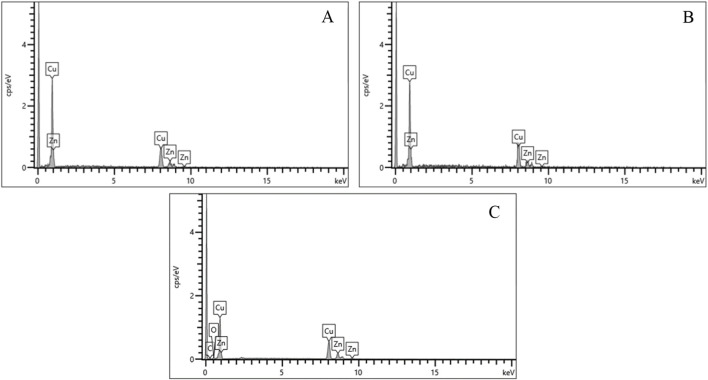
Energy-dispersive X-ray (EDS) spectra of the elemental composition of cartridge cases from **(A)** Fiocchi NTA **(B)** ADCOM ammunition **(C)** NATO ammunition.

**FIGURE 7:**
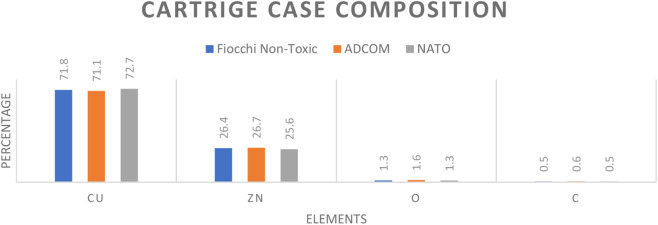
Casing composition of Fiocchi NTA, ADCOM and NATO ammunitions.

Despite differences in bullet composition, the casing alloys were consistent across all three ammunitions. EDS spectra (Figure 6) and quantitative results (Figure 7) showed that all cases were primarily composed of copper (Cu) and zinc (Zn), characteristic of brass. This uniformity suggests that cartridge case residues have limited discriminatory value in forensic analysis compared to primers and bullets.

**FIGURE 8:**
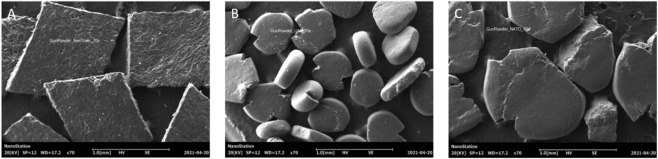
Gunpowder SEM image 70x of **(A)** Fiocchi NTA **(B)** ADCOM ammunition **(C)** NATO ammunition.

**FIGURE 9:**
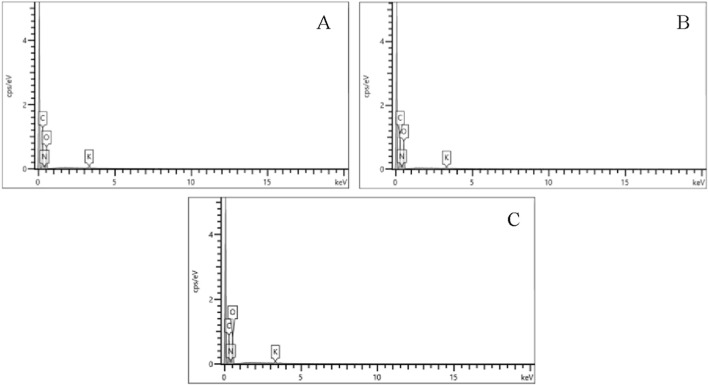
Energy-dispersive X-ray (EDS) spectra of the elemental composition of gunpowder from **(A)** Fiocchi NTA **(B)** ADCOM ammunition **(C)** NATO ammunition.

**FIGURE 10:**
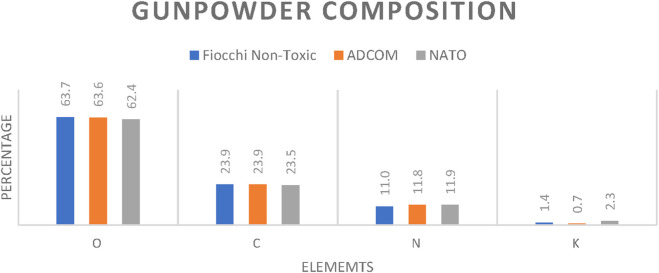
Gunpowder composition of Fiocchi NTA, ADCOM and NATO ammunitions.

Morphological variation was observed in the gunpowder grains (Figure 8), but elemental composition remained uniform across Fiocchi, ADCOM, and NATO (Figure 9; Figure 10). All powders consisted predominantly of oxygen (O), carbon (C), and nitrogen (N), consistent with nitrocellulose and nitroglycerin, and contained small but detectable amounts of potassium (K), indicative of potassium nitrate (KNO_3_). The similarity highlights that gunpowder chemistry is not the source of major differences in GSR toxicity, reaffirming that primer formulations play the decisive role.

Primer mixture composition analysis of Fiocchi NTA ammunition revealed the unexpected presence of lead (∼5–10%), although it is marketed as heavy metal-free ammunition (Table 2). This concentration, however, is significantly lower than the lead content in typical toxic ammunition (∼21–42%), as shown in Tables 3, 4. Additionally, the Fiocchi NTA ammunition included elements such as silicon, magnesium, chlorine, and iron, commonly found in sand, a known component of NTA ammunition (Black et al., 2021). Some Fiocchi NTA samples exhibited typical toxic GSR composition (Pb-Ba-Sb), whereas others displayed a composition aligned with non-toxic GSR elements (Cu, K, Al, Zn, Si, Mg, Fe), indicating a hybrid composition aiming to reduce overall toxicity (Table 2). A limitation of this study is that only a single lot per ammunition type was examined, which may not fully capture variability across different manufacturing batches; nonetheless, the findings remain consistent with international reports of residual lead in certain “non-toxic” primers.

**TABLE 2:** Primer residue composition of Fiocchi NTA using SEM/EDS.

Trial\Element	Pb	Ba	Sb	O	C	S	Cu	K	Al	Zn	Si	Mg	Fe	Cl
1	10.3	24.8	15	25.3	9.6	3.6	2.9	7.9	​	​	​	​	​	0.6
2	8.4	19.9	15.6	28.6	10.7	4.1	​	6	3	​	3.6	​	​	​
3	6.8	15.4	8.1	32	12	2.5	9.3	6.2	1.8	3.3	2.7	​	​	​
4	5.3	9.5	5.4	47.5	17.8	1.7	4.8	5.1	1.3	​	1.5	​	​	​
5	​	​	​	45.8	17.2	24	2.5	5.6	2	​	3	​	​	​
6	​	​	​	45.8	17.2	24	2.5	5.6	2	​	3	​	​	​
7	​	​	​	43.8	16.4	​	2.9	1.8	​	​	20.8	14.3	​	​
8	​	​	​	27.4	10.4	​	4.2	8.3	1.4	​	14.3	16.9	16.3	0.8
9	​	1.3	​	43.8	16.5	​	6.8	17.4	4	2.4	6.3	0.9	​	0.6
10	11.6	30.8	22.1	20	7.5	7.9	​	​	​	​	​	​	​	​

**TABLE 3:** Primer residue composition of ADMOC ammunition using SEM/EDS.

Trial\Element	Pb	Ba	Sb	O	C	S	Cu	K	Al	Zn	Si	Mg	Fe	Cl
1	29.4	21	17.4	13.4	5	6.2	1.7	3.9	2	​	​	​	​	​
2	28	14.7	12.1	22.6	8.5	4.7	3.1	4.8	1.5	​	​	​	​	​
3	27.7	21	16.2	15.6	5.9	4.6	3.9	4	1.2	​	​	​	​	​
4	22.9	19.9	15.3	18.4	6.9	5.6	7.5	2.7	0.8	​	​	​	​	​
5	41.7	11.1	11.1	12.8	4.8	5.6	7	4.8	1.1	​	​	​	​	​
6	38.7	7.2	14.1	18	6.8	6.1	3.1	4.8	1.3	​	​	​	​	​

**TABLE 4:** Primer residue composition of NATO ammunition using SEM/EDS.

Trial\Element	Pb	Ba	Sb	O	C	S	Cu	K	Al	Zn	Si	Mg	Fe	Cl
1	29.1	29	16.9	9.4	3.5	6.5	3.4	​	​	2.2	​	​	​	​
2	25.7	22.9	14.6	17.2	6.5	5.4	5.2	​	​	2.6	​	​	​	​
3	21.9	7.9	8.3	41.6	15.6	4.7	​	​	​	​	​	​	​	​
4	24.4	31.6	23	10.4	3.9	6.7	​	​	​	​	​	​	​	​
5	21	28.7	19	18.8	7.1	5.5	​	​	​	​	​	​	​	​
6	23.4	11.2	14.3	33.2	12.5	5.5	​	​	​	​	​	​	​	​

**FIGURE 11:**
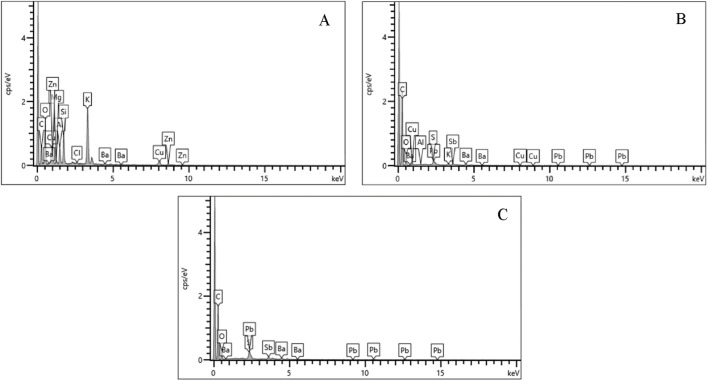
Energy-dispersive X-ray (EDS) spectra of the elemental composition of primer residue from **(A)** Fiocchi NTA **(B)** ADCOM ammunition **(C)** NATO ammunition.

By contrast, ADCOM and NATO primers followed the traditional pattern of Pb, Ba, and Sb dominance, with EDS spectra (Figure 11) showing strong peaks corresponding to lead styphnate/lead azide (Pb), barium nitrate (Ba), and antimony trisulfide (Sb). The high concentrations of these elements explain the greater number of characteristic Pb-bearing particles observed in post-discharge samples.

Taken together, these results demonstrate that bullet and casing compositions show limited variability, whereas primer chemistry provides the most reliable basis for differentiating toxic versus hybrid non-toxic ammunition. The detection of residual lead in Fiocchi NTA is especially significant, as it highlights the limitations of “lead-free” commercial claims and highlight the importance of direct forensic verification rather than reliance on manufacturer labelling.

### Ammunition’s GSR type comparison

**FIGURE 12:**
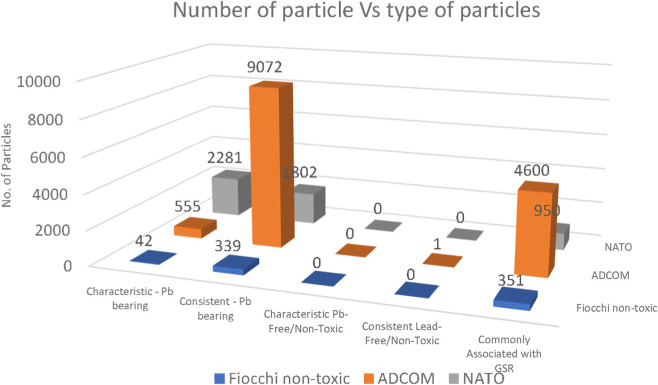
Number of GSR particles for all the classifications collected from both hands of Fiocchi, ADCOM and NATO ammunitions (n = 5 shots).

Gunshot residue (GSR) particles from Fiocchi NTA, ADCOM, and NATO ammunition were characterized using the automated SEM/EDS search program. All reported results correspond to the five-shot trials, as this condition provided the most representative particle distributions and allowed clearer assessment of variation. The one and three shot trials were retained solely to evaluate the effect of shot number on residue production. Quantitative comparison (Figure 12) revealed clear differences between the three ammunition types. NATO ammunition generated the highest number of characteristic Pb-bearing particles (often exceeding several thousand per sample), followed by ADCOM, while Fiocchi NTA consistently produced the lowest particle counts. This hierarchy directly reflects the primer compositions reported in Tables 2–4, where NATO and ADCOM primers contained substantially higher Pb (21%–42%) compared to Fiocchi NTA (5%–10%). The strong correlation between primer chemistry and post-discharge particle yield emphasizes the dominant role of primer formulation in GSR production.

**FIGURE 13:**
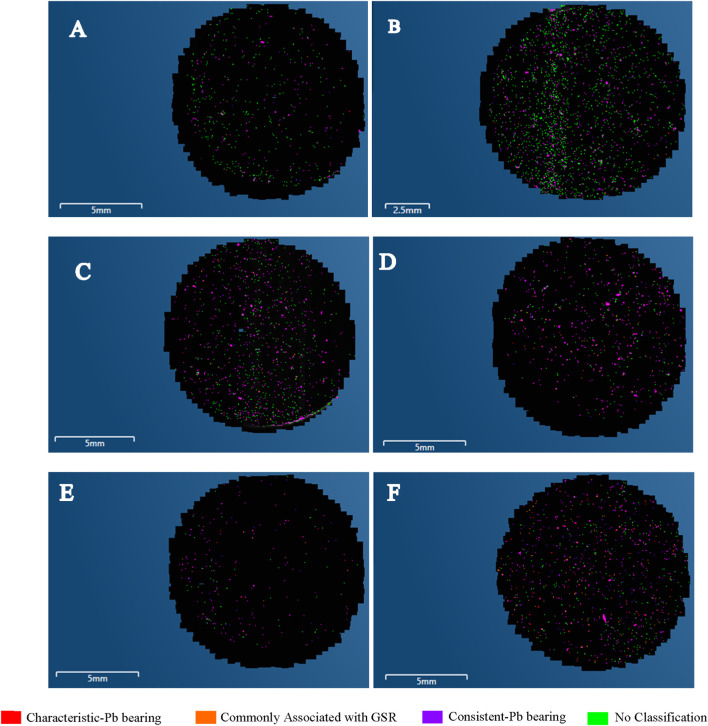
GSR particle distribution maps acquired immediately after discharge (time = 0) following five shots. **(A)** Fiocchi ammunition–Left hand, **(B)** Fiocchi ammunition–Right hand, **(C)** ADCOM ammunition–Left hand, **(D)** ADCOM ammunition–Right hand, **(E)** NATO ammunition–Left hand, **(F)** NATO ammunition–Right hand. Color coding as shown in the figure.

Particle mapping data (Figure 13) further supports this trend. For NATO and ADCOM, both left and right hand samples showed dense clustering of characteristic Pb-bearing and consistent Pb-bearing particles, with the right hand displaying higher deposition due to its proximity to the ejector port. In contrast, Fiocchi maps revealed sparse deposition and larger proportions of particles categorized as “commonly associated with GSR” or “unclassified,” reflecting the reduced Pb content and hybrid elemental composition of this ammunition type.

Despite Fiocchi being marketed as “non-toxic,” no Pb-free or HMF particles were identified by the AZTEC GSR software in any of the five-shot trials (Figure 12). This apparent absence is not due to their non-existence, but rather the limitations of the ASTM E1588-20 classification scheme, which currently recognizes only two types of Pb-free characteristic particles. As demonstrated in recent studies (Bauer et al., 2016; Donghi et al., 2019), many newer formulations, especially hybrid NTA primers containing elements such as Al, Si, Zn, or Ti, fall outside the current automated classification and are therefore missed.

From a forensic perspective, these findings are significant. While traditional Pb–Ba–Sb residues remain readily detectable in NATO and ADCOM ammunition, Fiocchi NTA highlights a growing challenge: the risk of underreporting or false negatives when relying solely on existing classification schemes. The combination of low Pb levels and inadequate software recognition criteria means that hybrid or marketed “non-toxic” ammunition may still leave detectable residues, but their identification requires manual review or updated classification libraries.

### GSR particle size

**FIGURE 14:**
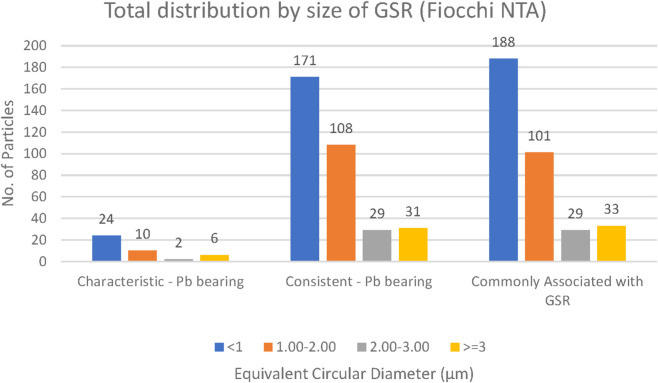
Total distribution of GSR particles size collected from both hands of Fiocchi NTA (n = 5 shots).

**FIGURE 15:**
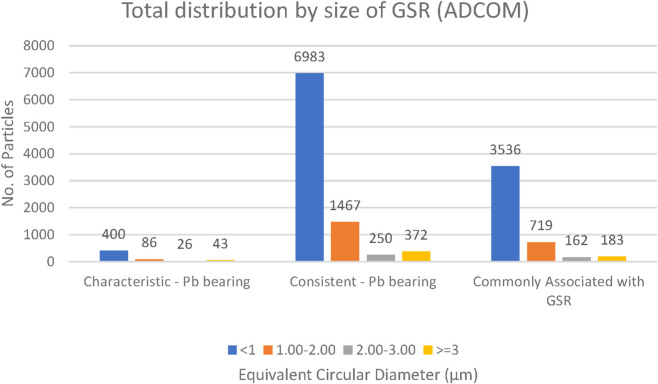
Total distribution of GSR particles size collected from both hands of ADCOM ammunition (n = 5 shots).

**FIGURE 16:**
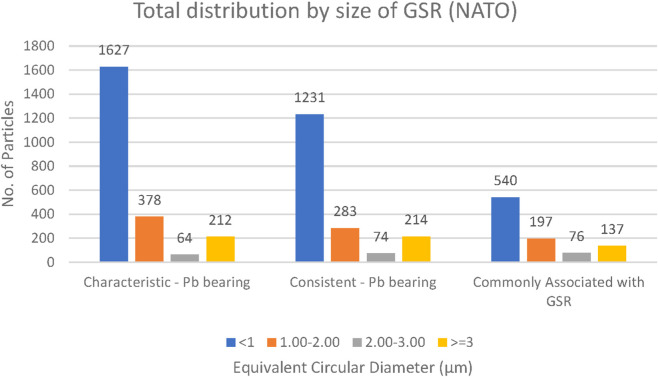
Total distribution of GSR particles size collected from both hands of NATO ammunition (n = 5 shots).

GSR particles were classified into four size categories: <1 μm, 1–2 μm, 2–3 μm, and >3 µm. Across all ammunition types (Fiocchi NTA, ADCOM, and NATO), the majority of particles measured less than 3 μm, with a clear predominance of submicron particles (<1 µm) (Figures 14–16). This trend is forensically significant because it provides a robust distinction from most consistent environmental or occupational particles, typically exceeding 100 µm in size (Phermpornsagul et al., 2020).

Closer comparison among ammunition types revealed that NATO and ADCOM samples yielded higher absolute counts of submicron particles compared to Fiocchi NTA, mirroring their higher lead content and primer-driven particle generation (Table 2–4). Fiocchi NTA, while producing fewer particles overall, still exhibited a similar size distribution pattern dominated by particles smaller than 1 µm. This suggests that primer chemistry influences the quantity but not the fundamental size distribution of generated GSR (Charles and Jonckheere, 2022; Li et al., 2024).

The predominance of particles below 3 µm also has practical implications for forensic detection. Smaller particles are more readily dispersed into the surrounding environment and can persist on surfaces or skin even after secondary transfers (Gorey et al., 2024), making them valuable markers for post-event analysis. Conversely, their small size also means they are more easily lost during routine post-shooting activities such as handwashing or wiping, which may explain variability in recovery rates across time intervals (Taudte et al., 2016; Werner et al., 2020).

### GSR collection time

**FIGURE 17:**
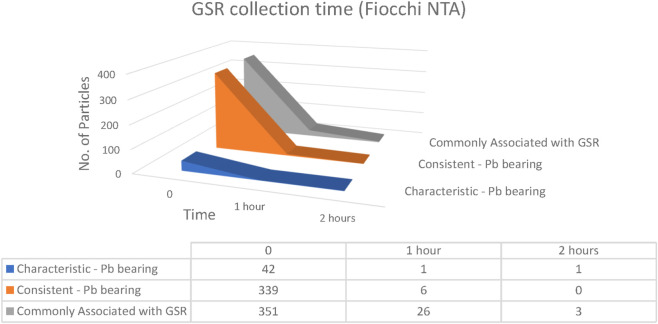
Number of particles as a function of elapsed time collected from both hands of Fiocchi NTA (n = 5 shots).

**FIGURE 18:**
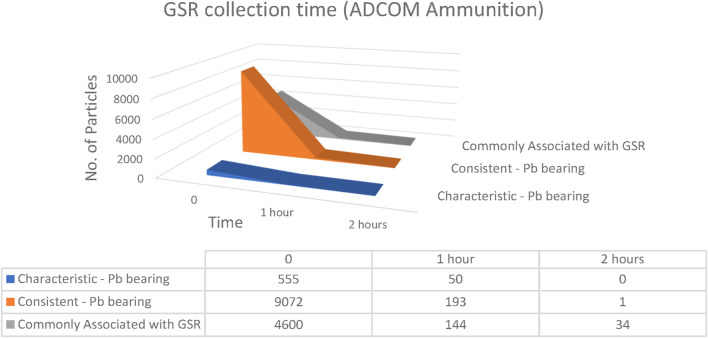
Number of particles as a function of elapsed time collected from both hands of ADCOM ammunition (n = 5 shots).

**FIGURE 19:**
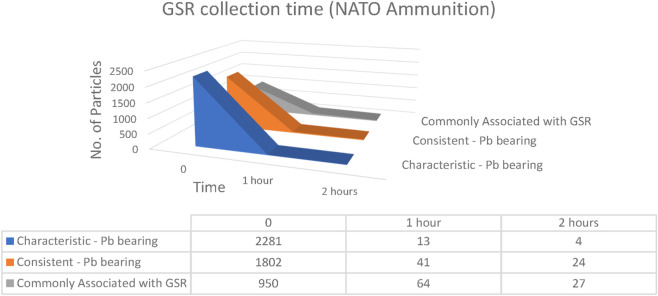
Number of particles as a function of elapsed time collected from both hands of NATO ammunition (n = 5 shots).

The time interval between firearm discharge and GSR collection is one of the most important variables influencing evidential recovery. Internationally, a sampling window of 4–6 h is often cited as acceptable (Charles et al., 2020), although prior studies have shown that particle numbers typically decrease within the first 1–3 h (Phermpornsagul et al., 2020). To evaluate short-term persistence, samples were collected at 0, 1, and 2 h post-discharge from both hands of volunteers using Fiocchi NTA, ADCOM, and NATO ammunition (Figures 17–19).

For Fiocchi NTA, relatively low particle counts were recorded, and a rapid decline was observed over time. At the time of discharge, 42 characteristic Pb-bearing particles, 339 consistent Pb-bearing particles, and 351 commonly associated particles were detected. After 1 h, these numbers decreased to 1, 6, and 26 particles, respectively, and by 2 h only 1 characteristic particle and 3 commonly associated particles remained detectable. This steep decline indicated that residues produced by Fiocchi NTA primers were less stable and more readily lost due to post-shooting activities such as handwashing or contact with laboratory surfaces (Rosengarten et al., 2021; Taudte et al., 2016; Werner et al., 2020).

In the case of ADCOM ammunition, much higher initial counts were measured. At discharge, 555 characteristic Pb-bearing particles, 9,072 consistent Pb-bearing particles, and 4,600 commonly associated particles were detected. After 1 h, these values had dropped to 50, 193, and 144 particles, respectively, while at 2 h only 1 consistent particle and 34 commonly associated particles were recorded, with no characteristic Pb-bearing particles remaining. The marked reduction again reflected the influence of physical activities performed by the shooters, which accelerated the loss of loosely attached residues (Rosengarten et al., 2021).

For NATO ammunition, the most robust and persistent profile was obtained. At time zero, 2,281 characteristic Pb-bearing particles, 1,802 consistent Pb-bearing particles, and 950 commonly associated particles were detected. After 1 h, these values decreased to 13, 41, and 64 particles, respectively. At 2 h, 4 characteristic particles, 24 consistent particles, and 27 commonly associated particles were still recorded. Unlike Fiocchi and ADCOM, which showed near-complete disappearance of characteristic particles within 2 h, NATO residues remained clearly detectable. The higher persistence of NATO particles suggested that primer chemistry played a dominant role, while variations in particle recovery were further influenced by the shooters’ activities during the post-discharge period (Brożek-Mucha, 2017; Charles et al., 2022).

Overall, the results demonstrated that maximum recovery occurred immediately after discharge for all ammunition types, followed by marked reductions over time. Residues from Fiocchi NTA diminished almost completely within 2 h, while ADCOM and NATO residues, although significantly reduced, remained detectable, with NATO showing the strongest persistence. From a forensic perspective, these findings indicate the critical importance of prompt sampling, while also demonstrating that residues produced by Pb-rich ammunition can still provide probative value up to 2 h post-discharge. The observed variations in particle recovery at each interval were attributed not only to elapsed time but also to the normal activities of the shooters, which significantly influenced the extent of residue loss (Taudte et al., 2016; Werner et al., 2020). During these waiting intervals, only normal low-intensity activities such as walking and sitting were permitted, while handwashing or contact with potentially contaminated surfaces was avoided. This approach was intended to reflect realistic post-shooting conditions without introducing artificial contamination.

### GSR number of shots effect

The influence of the number of shots on GSR particle recovery was evaluated by comparing samples collected from the right and left hands of volunteers following 1, 3, and 5 discharges of Fiocchi NTA, ADCOM, and NATO ammunition (Table 5). In principle, an increased number of shots would be expected to correlate with a greater number of recoverable GSR particles, owing to the higher cumulative emission of waste. However, the results demonstrated that this relationship was not consistently observed across ammunition types, in agreement with earlier reports highlighting the stochastic nature of GSR deposition (Brożek-Mucha, 2017; Taudte et al., 2016).

**TABLE 5:** Number of shots effect on GSR particles counts collected from both hand of Fiocchi, ADCOM and NATO ammunitions.

Ammunition type	# Of shots	Characteristic - Pb bearing	Consistent - Pb bearing	Characteristic Pb-Free/Non-Toxic	Consistent lead-Free/Non-toxic	Commonly associated with GSR	Total classified particles
Fiocchi NTA	1	81	361	0	4	248	694
	3	106	401	0	3	586	1,096
	5	42	339	0	0	351	732
ADCOM	1	64	305	0	0	276	645
	3	70	376	0	0	185	631
	5	555	9,072	0	1	4,600	14,228
NATO	1	619	2045	0	0	816	3,480
	3	299	2,300	0	5	1892	4,496
	5	2,281	1802	0	0	950	5,033

For Fiocchi NTA, particle recovery did not scale with the number of shots. After a single shot, 694 total classified particles were detected, including 81 characteristic Pb-bearing and 361 consistent Pb-bearing particles. Following three shots, the total increased modestly to 1,096 particles, yet the proportion of characteristic Pb-bearing particles remained low (106 particles). Unexpectedly, after five shots, the total count decreased to 732 particles, with only 42 characteristic Pb-bearing particles detected. This irregular pattern suggested that Fiocchi residues were deposited inconsistently, likely due to the hybrid primer chemistry generating lower and less stable particle yields, which were more susceptible to loss through handling activities (Rosengarten et al., 2021; Werner et al., 2020).

For ADCOM ammunition, the trend was more aligned with theoretical expectations. After one shot, 645 total particles were detected, including 64 characteristic Pb-bearing particles. After three shots, the total remained relatively similar (631 particles), but following five shots, particle recovery increased dramatically to 14,228 total particles, with 555 characteristic Pb-bearing particles and 9,072 consistent Pb-bearing particles. This sharp increase highlighted the effect of repeated discharges in generating dense clouds of Pb–Ba–Sb residues, which resulted in more consistent deposition on the shooter’s hands.

For NATO ammunition, particle counts again varied with shot number but not in a strictly linear manner. After one shot, 3,480 total particles were detected, including 619 characteristic Pb-bearing particles. Interestingly, after three shots, the total was lower (4,496 particles) than might have been expected, with 299 characteristic particles recorded. However, after five shots, the total increased to 5,033 particles, including a substantial 2,281 characteristic Pb-bearing particles. The recovery pattern thus demonstrated variability between replicates but still showed that higher shot numbers, particularly five discharges, yielded markedly greater counts of characteristic residues compared with single- or three-shot scenarios.

Overall, the results demonstrated that while ADCOM and NATO generally produced larger particle numbers with increasing shots, Fiocchi NTA showed a highly inconsistent relationship between shot number and particle recovery. These findings highlight that the number of discharges is only one factor influencing GSR deposition, and that particle recovery is also dependent on primer chemistry, deposition dynamics, and the activities of the shooter in the post-discharge period (Charles et al., 2022; Taudte et al., 2016). From a forensic standpoint, this variability reinforces the importance of interpreting GSR results with caution: a low particle count does not necessarily exclude firearm discharge, particularly when non-toxic or hybrid ammunition is involved.

### GSR collection region effect

Since all volunteer shooters were right-handed, they consistently positioned their dominant hand above the non-dominant, partially shielding the latter from GSR deposition. Consequently, significantly more GSR particles were recovered from the right hand compared to the left for all ammunition types (Table 6). Another influencing factor is the firearm type utilized, the Browning Hi-Power pistol, which features an ejector port on its right side. Prior research confirms that the ejector port side of semi-automatic pistols generally yields a higher GSR deposition on the hand proximal to it, even though both hands are exposed (Werner et al., 2020).

**TABLE 6:** Collection area effect on GSR particles of Fiocchi, ADCOM and NATO ammunitions (n = 5 shots).

Bullet type	Collection area (R-right/L-Left) hand	Characteristic - Pb bearing	Consistent - Pb bearing						Commonly associated with GSR		
​	​	Pb Sb Ba	Pb Ba Ca Si	Ba Ca Si	Sb Ba	Pb Sb	Ba Al	Pb Ba	Pb	Sb	Ba
Fiocchi NTA	R	27	0	4	47	21	33	37	126	27	93
	L	15	0	3	26	6	130	32	20	15	70
ADCOM	R	459	0	5	536	113	4,436	521	83	41	2,823
	L	96	0	2	232	29	3,042	156	85	146	1,422
NATO	R	2021	0	0	1,504	38	20	12	302	288	141
	L	260	0	0	218	8	1	1	175	27	17

For Fiocchi NTA, the right hand yielded a total of 415 classified particles, including 27 characteristic Pb–Sb–Ba particles, 47 Sb–Ba particles, and 126 Pb particles. In comparison, only 302 total classified particles were detected on the left hand, with 15 characteristic Pb–Sb–Ba particles and lower counts across other categories. The reduced particle recovery from the left hand reflected both shielding effects and the lower emission levels associated with Fiocchi’s hybrid primer composition.

For ADCOM ammunition, the inconsistency between hands was more pronounced. The right hand showed 7,476 total classified particles, including 459 characteristic Pb–Sb–Ba particles, 536 Sb–Ba particles, and 2,823 Ba particles. In contrast, the left hand yielded only 5,070 total classified particles, including 96 characteristic particles and 1,422 Ba particles. The markedly higher deposition on the right hand supported the strong influence of firearm mechanics and primer chemistry on residue distribution.

For NATO ammunition, the most substantial difference was observed. The right hand exhibited 4,327 total classified particles, including 2,021 characteristic Pb–Sb–Ba particles and 1,504 Sb–Ba particles. The left hand, by comparison, showed only 699 total classified particles, with 260 characteristic particles and 218 Sb–Ba particles. This nearly six-fold difference highlighted both the directional discharge of residues from Pb-rich NATO primers and the mechanical influence of the right-sided ejector port.

Taken together, these results confirmed that GSR deposition was significantly greater on the right (dominant) hand than on the left across all ammunition types. The effect was strongest for NATO, intermediate for ADCOM, and weakest for Fiocchi NTA, mirroring the underlying primer chemistries. From a forensic perspective, these findings emphasize the necessity of sampling both hands during casework: although right-hand samples consistently yield higher particle counts, left-hand samples still provide supportive residues that may strengthen evidential interpretation (Brożek-Mucha, 2017; Charles et al., 2022; Werner et al., 2020).

## Conclusion

This study evaluated three types of 9 mm ammunition, Fiocchi NTA, ADCOM, and NATO, using SEM/EDS analysis in accordance with ASTM E1588-20 standards. Toxic traditional ammunitions (ADCOM and NATO) consistently produced detectable GSR particles under all tested conditions, emphasizing their forensic reliability. In contrast, Fiocchi NTA, marketed as non-toxic, showed measurable Pb content (∼5–10%) in primer material, indicating residual toxicity and aligning with previous studies that some “non-toxic” formulations still rely on Pb for ignition stability. Moreover, no Heavy-Metal-Free (HMF) GSR particles were detected for Fiocchi NTA due to the limitations of the current ASTM criteria. GSR persistence was demonstrated up to 2 h post-discharge, though strongly influenced by post-shooting activities. Collectively, these findings highlight the limitations of existing ASTM classification, the challenges posed by hybrid primer formulations, and provide critical reference data to strengthen global knowledge on GSR characterization.

Given the continuous emergence of new ammunition formulations, particularly NTAs with diverse chemical profiles, future research should prioritize extensive characterization of various ammunition brands and types. Establishing a comprehensive database and refining existing classification frameworks are essential steps toward enhancing forensic capabilities. Such advancements will significantly aid forensic investigations and judicial processes by improving the precision of linking suspects to shooting incidents.

## Data Availability

The original contributions presented in the study are included in the article/supplementary material, further inquiries can be directed to the corresponding author.

## Appendix

**TABLE A1:** Experimental design matrix for gunshot residue (GSR) sampling, showing ammunition type, number of shots, sampling time post-discharge, and volunteer identifiers.

Trial ID	Ammunition type	Number of shots	Sampling time-post discharge (hours)	Volunteer ID
1	ADCOM	1	0	V-01
2	ADCOM	1	1	V-05
3	ADCOM	1	2	V-12
4	ADCOM	3	0	V-01
5	ADCOM	3	1	V-06
6	ADCOM	3	2	V-08
7	ADCOM	5	0	V-01
8	ADCOM	5	1	V-07
9	ADCOM	5	2	V-13
10	NATO	1	0	V-01
11	NATO	1	1	V-08
12	NATO	1	2	V-14
13	NATO	3	0	V-01
14	NATO	3	1	V-03
15	NATO	3	2	V-15
16	NATO	5	0	V-01
17	NATO	5	1	V-04
18	NATO	5	2	V-14
19	Non-toxic FIOCCHI	1	0	V-01
20	Non-toxic FIOCCHI	1	1	V-02
21	Non-toxic FIOCCHI	1	2	V-09
22	Non-toxic FIOCCHI	3	0	V-01
23	Non-toxic FIOCCHI	3	1	V-03
24	Non-toxic FIOCCHI	3	2	V-10
25	Non-toxic FIOCCHI	5	0	V-01
26	Non-toxic FIOCCHI	5	1	V-04
27	Non-toxic FIOCCHI	5	2	V-11

Two separate hand samples (right and left) were collected for each experimental trial, yielding a total of 54 samples across 27 trials.
